# The Committee on American Medical Colleges

**Published:** 1881-10

**Authors:** 


### THE COMMITTEE ON AMERICAN MEDICAL COLLEGES.

Reported to the American College Association that having procured and carefully examined sixty-four announcements of different colleges for the year 1880-1, they find that five colleges do not require of candidates for graduation evidence that they have studied medicine three years; two colleges do not require attendance upon two courses of lectures; six colleges give the degree of M. D. to students who have attended their last course elsewhere; in four colleges the regular term is less than twenty weeks; five colleges give beneficiary tickets irrespective of the limitations prescribed by the American Medical College Association; two colleges grant Ad Eundum degree on terms other than those required by the Association, viz., on simple examination on the practical branches; three colleges give their lectures during the evening.

*Summary.*—Sixteen colleges violate some one or more of the requirements of the American Medical College Association.

Last year an examination of about the same number of catalogues exhibited the fact that thirty-five failed to show that they conformed to all the requirements of the American Medical College Association.

Forty-eight candidates were found to conform to the above requirements. This shows a marked improvement. Still more care in the preparation of the several announcements would place several of the sixteen in the conforming list.

It is proper to add that in all the essential elements of a medical college, a very considerable number of colleges (twenty-two) surpass the requirements of the Association. They require one or more of the following desiderata of the best medical college, viz.: Nine months yearly attendance; preliminary examination; three terms instead of two; real clinical work under the personal direction of a sufficient number of competent teachers. Thus, there are sixteen of sixty-four colleges behind the requirements of our Association, and twenty-two ahead of them in one or more points mentioned above.

